# Aspects of the owning/keeping and disposal of horses, and how these relate to equine health/welfare in Ireland

**DOI:** 10.1186/2046-0481-64-11

**Published:** 2011-09-21

**Authors:** Joseph A Collins, Alison Hanlon, Simon J More, Patrick G Wall, Vivienne Duggan

**Affiliations:** 1 Veterinary Sciences Centre, School of Veterinary Medicine, University College Dublin, Belfield, Dublin 4, Ireland; 2 School of Public Health, Physiotherapy and Population Science, University College Dublin, Belfield, Dublin 4, Ireland

**Keywords:** Horse, Welfare, Disposal, Ireland

## Abstract

**Background:**

Ireland has long been renowned as a major centre for the breeding, rearing and keeping of horses. Since 2007, however, there has been increasing concern for horse health and welfare standards, and links between these concerns and the structures, governance and funding of the Irish equine industries have been reported. This paper addresses two central issues: firstly the local governance of, trade in and disposal of unwanted horses; and secondly mechanisms employed to improve standards of care given to horses owned by certain communities.

**Method:**

Primary information was gathered through visits to horse pounds run by and on behalf of Local Authorities, to social horse projects, to horse dealer yards, ferry ports, horse slaughter plants and knackeries.

**Results:**

The approach adopted by members of a given group, e.g. ferry ports, is described and differences are highlighted, for example in how different Local Authorities implement the Control of Horses Act of 1986, and how the choice, for example, of disposal route affects the standard of animal welfare.

**Conclusions:**

There is a pressing need for a more centrally mandated and uniformly applied system of governance to safeguard the health and promote the keeping of horses to a higher welfare standard in Ireland. Fundamental to an understanding of why there is insufficient oversight of the keeping and proper disposal of horses is the lack of a comprehensive, integrated system for the registration, identification and tracing of equidae in Ireland.

## Background

Ireland has long been a major producer of horses of all types for the domestic market and for export abroad, ranking among the largest producers of Thoroughbred horses in Europe during the recent decade [1]. With an estimated 27.5 sport horses per thousand people it is the most densely sport horse populated country in Europe [2]. Links between the structures, governance and funding of the Irish equine industries and potential concerns for equine welfare have already been reported [3]. These authors also reported upon the perception of equine welfare [4,5] and on the welfare of horses on farms in Ireland [6]. The key issues to emerge from this work as drivers for poor welfare standards were problems with unwanted horses, especially the trade (most particularly via fairs and dealers) and disposal of horses by an owner/keeper when he/she no longer considered them fit for purpose.

The level of production of horses in Ireland has historically exceeded the domestic need and a variety of routes of removal of horses from the owned live Irish horse population have long existed. These include sale (including privately via sales companies, dealers and to slaughter plants); surrender to animal welfare charities for re-homing; abandonment; burial or disposal of carcases via knackeries; and export predominantly via ferry ports.

The Control of Horses Act was enacted in 1996 in response to a perceived problem with unwanted and straying horses, especially in urban areas. The legislation was designed to deal with horses being kept on local authority land without permission, horses being exercised in a manner which interfered with other amenity or land users (for example, on public beaches during the summer months), and the keeping of horses in inappropriate locations (for example, urban high density housing units), by persons with insufficient resources (for example, to house and feed horses according to their needs). Powers of enforcement were vested in the Local Authorities [7].

One mechanism for addressing poor standards of care of horses owned by inner city communities has been the 'social horse projects', which have been created in Ireland over the past decade. In most cases, these projects developed from informal community initiatives to facilitate the keeping of horses by inner city communities. In other cases, the prime driver was a desire (by agencies) to engage with defined communities using horses as an enabling mechanism for other social goals.

The aim of this paper is twofold. Firstly to review the management structures for dealing with unwanted or stray horses and describe routes of horse trading and disposal. Secondly to review mechanisms to improve responsible horse ownership amongst certain communities through schemes such as the 'social horse projects'.

## Methods

### Trade in and disposal of horses

#### Stray horses and The Control of Horses Act, 1996

Three horse pounds were selected for inclusion in this study, on the basis of geographical spread and significant difference in management structure: direct management by Louth County Council in the North East, by sub-contract from Cork County Council in the South West, and by private operators under the supervision of Kilkenny County Council in the South East. These pounds manage seized horses, pending payment of a reclaim fee. Each facility was assessed during a site visit, including a review of physical facilities and equipment, an examination of written records of the throughput of horses (where available) and interviews with staff members.

#### Horse slaughter plants (Abattoirs)

Until mid 2010, there were three abattoir facilities in the Republic of Ireland (ROI) licensed to slaughter horses for human consumption, and one in Northern Ireland which had suspended operations. Each of the three facilities (in Counties Kildare, Kilkenny and Limerick) that were actively engaged in the horse slaughter trade was visited. The physical facilities and methods for horse slaughter were reviewed, and members of staff were interviewed.

#### Category 2 Plants (Knackeries)

Plant operators of approved Category 2 Plants and sub-contractors in ROI were contacted in September 2007 by telephone. Each operator was asked to consult their records and provide details of the numbers of horse carcases handled at their facility during the past twelve months. Two sample knackeries were visited in 2009 to assess the facilities and disposal procedures.

#### Horse dealers

Visits were conducted to the farms of five known horse dealers in four counties (two in the Republic of Ireland and two in Northern Ireland). Dealers were identified by horse slaughterers, transporters, portal inspectors, veterinary groups, horse sales vendors and animal welfare societies. Information was gathered during inspection of facilities and interviews, and photographs were taken of facilities and horses.

#### Ferry ports

Contacts were made with the portal veterinary inspector at each ferry port capable of the import and export of live horses from the island of Ireland. Visits were conducted to those ports with records of horse throughput to view the facilities, interview staff and study/collect records. These ports were:

• Larne and Belfast (both Co. Antrim)

• Dublin and Dún Laoghaire (both Co. Dublin)

• Rosslare (Co. Wexford)

### Social horse projects

Social horse projects were investigated in the Dublin and Kilkenny areas. In Dublin, these were the Cherry Orchard Equine, Education and Training Centre, the Fettercairn Youth Horse Project and the Meakestown Equestrian Facility, each with established equestrian facilities. In Kilkenny, the Kilkenny Community Action Network (KCAN) project focuses on local horse-keeping groups through the medium of horses but without central equestrian facilities.

The following protocol was adopted for all four projects: an inspection of facilities and interviews with staff and clients. Further information was elicited through a study of media reporting. In addition, visits were made to the Smithfield horse fair, a monthly equestrian event with links to the three social horse projects in the Greater Dublin area.

## Results

### Trade in and disposal of horses

#### Horse pounds and the Local Authorities

Each of the three horse pounds visited was in a rural setting. Each employed security such as lights, razor-wire, high fences, CCTV, guard dogs, lock-down at night, security patrols and intruder alarms and can be differentiated as follows:

• *Louth*. This pound was a purpose-built, managed and serviced premises with direct supervision by the Local Authority veterinarian. There were horse stables and horse transport equipment; in addition there were kenneling facilities for impounded dogs and cats. The pound occasionally took in animals at the request of neighbouring Local Authorities in North Leinster/Ulster.

• *Cork*. This pound was operated as a sub-contracted private business, employing private veterinary services for animal treatments. The pound was regularly inspected by the Local Authority veterinarian. Animals were collected at the request of several Local Authorities in Munster including Cork and Limerick City and County Councils.

• *Kilkenny*. This pound was privately owned and managed, gathering horses from a wide geographical area (predominantly Leinster and Connacht) at the behest of multiple Local Authorities. It employed private veterinary services as needed for animal care.

Each pound operated under the direction of one or more Local Authorities under powers defined by the Control of Horses Act, 1996 which permits them to define (by means of bye-laws) both 'Exclusion Areas' where the presence of horses is not permitted and the resource inputs which an owner/keeper is required to provide before a license will be granted to keep horses in a designated 'Control Area'. Funding was provided centrally by the Department of Agriculture, Food and Fisheries (DAFF). Local Authorities varied in how they defined areas for special consideration in regards to the keeping of horses. For example, Limerick City Council designated all of the area under its control a 'Control Area', but seemingly employed its powers to authorise the seizure and impounding of horses only sporadically. Louth County Council defined 'problem' areas as Control Areas (for example, regions of commonage, public beaches or urban zones where horses might compete with other grazing species, leisure users or dwellers, respectively) and instigated a systematic and rigorous set of requirements for the licensing, exercising and keeping of horses in that area.

Most County Councils had not sought to develop and maintain their own fully functional horse pound, instead outsourcing its collection and impounding functions. Under this template, the Local Authority authorized the seizure (by sub-contractors) of horses deemed to be in contravention of its bye-laws to be kept at the pound, microchipped for recording purposes and released on production of a receipt-of-payment-of-a penalty issued by the Local Authority to a licensed person. Unclaimed horses, and those repeatedly seized, could be otherwise disposed of. Louth County Council had developed an alternate template. Authorised officers (local authority veterinarian and inspectors) patrolled the 'Control Area' in a marked horse-transport vehicle, creating a visible presence and actively engaging with the horse-owning/keeping community. Staff offered a service (the identification and licensing of horses) to owners who showed a willingness to comply with local bye-laws, and otherwise impounded horses where necessary - either in the public interest and/or to show that the legislation has teeth. Louth Local Authority staff expressed the view that this interaction led to an improvement in compliance with the law, a culture change over time and to a reduction in the incidence of serious problems with irresponsible horse keeping.

#### Horse slaughter plants (Abattoirs)

The three active slaughter horse slaughter facilities in Ireland in 2010 differed in location, supervision and species processed, as follows:

• Co. Kilkenny: a long-established business processing horses on average two days per week (with cattle and sheep on the other days), supervised by DAFF veterinary inspectors;

• Co. Limerick: a Local Authority supervised plant processing a range of animal species according to market requirements and commenced horse slaughter from early 2009; and

• Co. Kildare: a DAFF supervised, re-commissioned, purpose-built horse slaughter plant that recommenced the slaughter of horses in late 2009 under new management.

In each facility, the slaughter process itself was considered to be carried out in a satisfactory manner with due regard to the principles of horse handling and humane slaughter [8]. Purchasing staff reported that that they had no current difficulty sourcing horses for slaughter but that there were greater difficulties with sourcing 'suitable horses for the human food chain'. Horse identification, conformation/body condition and health/drug history are the main criteria for selecting horses to enter the food chain. Ineligible horses, usually procured as part of a job-lot, were disposed of through the knackery system at a loss to the plant operator and typically included:

• Foals and yearlings;

• Lightweight athletic types such as young, racing-fit Flat Thoroughbreds, which produce overly lean carcases;

• Undernourished and debilitated horses which produce poor quality carcases at best suitable only for the low value, processing end of the market (with poor financial returns).

• Undocumented horses and those with documents signed as 'Excluded from the food chain' for reasons of owner choice or medication history.

The horse slaughter business was considered by staff to have changed in four significant ways in the recent past:

1) Horses have become an expensive luxury to many. Increasingly, those in the horse industries wish or need to dispose of surplus horses in a cost efficient manner.

2) There is an increasingly anthropomorphic and moralistic depiction of unwanted horses by the media, casting the equine industries in an unfavourable light.

3) More operators have entered the horse slaughter trade, competing for limited markets.

4) There is a higher public awareness of the trade.

#### Category 2 plants (Knackeries)

Category 2 plants (knackeries) are licensed, in the Republic of Ireland, to collect horse carcases not intended for human consumption, and are not currently required to submit records to a central database. Horse identification documents are not sought, nor collected or returned to the Horse Passport Issuing Authority for recording of the death of the horse on a database. The annual throughput of horse carcases reported by plant operators is shown in Table 1. The total estimated number of horse carcases processed by this route in the period examined was 1,973. More than half (53%) of the plants processed fewer than 20 horses in that period.

**Table 1:** The estimated number of horse carcasses disposed of during the preceding 12 months by each of 39 Category 2 knackery plants (denoted as per the county of location), as reported in September 2007

Province and number of horses	Munster	**No**.	Leinster	**No**.	Connacht	**No**.	Ulster	**No**.
	Cork	120	W.Meath	330	Roscommon	24	Cavan	18

	Limerick	25	Meath	300	Galway	12	Cavan	18

	Clare	14	Laois	13	Galway	42	Cavan	36

	Tipperary	50	Kilkenny	8				

	Tipperary	6	Carlow	12				

	Tipperary	13	Meath	110				

	Tipperary	6	Louth	18				

	Waterford	7	Wexford	100				

	Tipperary	12	Carlow	12				

	Cork	50	Wicklow	340				

	Tipperary	18	Laois	24				

	Cork	20	Meath	6				

	Tipperary	70	Longford	48				

	Tipperary	0	Offaly	12				

	Cork	3	Meath	16				

	Cork	20						

	Tipperary	50						

	Cork	10						

No. of counties and total in the province	5 of 6		10 of 12		2 of 5		1 of 3	

Total horse numbers		474		1349		78		72

#### Horse dealers

The facilities and resource inputs on view at horse dealer yards varied in standard. In each case there were 'front-of-house' stables for public viewing. The 'front-of-house' horses were generally kept individually stabled in circumstances considered typical of Irish equestrian facilities. Holding yards were subsequently viewed, where entry was by invitation only. Here horses were kept in groups in barns, outdoor pens and fields, and fed on large bale hay/haylage. Horses were held here and further assessed for suitability for onward trade as riding/driving/breeding animals or for slaughter. There were horse-transport lorries on view capable of holding up to 18 horses. In some instances, these had GB license plates.

There were often horses of moderate (acceptable) quality and welfare state on view in the more public facilities. However, at other holding facilities, lame, injured, ill and thin horses were viewed which were reported as being intended for slaughter. Circumstances did not allow the viewer to intervene in these instances but simply to observe and gather information. Dealers openly admitted that they did not necessarily seek horse identification documents (in contravention of the law) when sourcing horses as they could apply to a Horse Passport Issuing Authority of their choice for a new set.

#### Ferry ports

In no port were horses routinely unloaded, inspected to ascertain their health and welfare status, or cross-checked with regard to their travel or identification documents. At most ports, the number of horses in the shipment was noted and referenced to the number of identification documents offered by the shipper. Ferry ports have begun to record the detail of proffered information, by means of listing document and/or microchip numbers or photocopying documents. In one port, the introduction of this practice led to the discovery that a known shipper-for-slaughter was repeatedly reusing horse identification documents for successive shipments. Larne is currently the only port on the island of Ireland with facilities for the inspection of horses in lorries by means of a gantry and viewing platform and unloading facilities that could be used to inspect horses pre-export or import. Information was gathered regarding the throughput of horses per month where records exist and is summarised in Table 2. No such records exist for the Dublin ports. There was no information recorded at ferry ports concerning the purpose for which horses were exported or imported, or how many individual horses traveled both in and out via any port. There was no system to trace the movement of individual horses on and off the island of Ireland.

**Table 2:** The combined number of horses exported and imported per calendar year between January 2006 and December 2009, inclusive via Larne, Belfast and Rosslare ports combined

Year	2006	2007	2008	2009
Export	9,762	9,975	9,630	9,496

Import	7,288	6,956	5,763	4,683

Net export	2,474	3,019	3,867	4,813

No records exist for the numbers moving through the Co. Dublin ferry ports during this time period.

### Social horse projects

#### Cherry Orchard Equine, Education and Training Centre

Based in Ballyfermot, a densely-populated area of west Dublin, this project commenced approximately ten years ago as a local community initiative in response to the commencement of The Control of Horses Act, 1996. Funding (for capital and current expenditure) was secured both centrally (DAFF, Department of Education, and Department of Enterprise, Trade and Employment) and locally (Dublin City Council). Based on interviews with staff, it seems that initially there was a perception by local groups that the Cherry Orchard initiative would provide an equestrian facility for the local community to house their horses and use the facilities at will and under local community direction. There was a sense (on all sides) that this would lead to little or no change in the local horse culture. However, the facility has evolved otherwise: the horses are owned and managed by the Centre, which provides subsidized, structured training to local groups. At the time of inspection, there were 28 stables, 25 microchipped horses/ponies, 5 hectares of grazing, and both indoor and outdoor riding facilities. Teaching sessions were conducted in equine skills - both riding and general horse husbandry - for locals, either individually or on referral from Dublin City Council, An Garda Síochána, and Youth or Disability Groups. In 2010, approximately 600 persons attended weekly courses at the centre raising education standards through FETAC modules or providing a path to a professional equestrian career, for example via RACE (the Racing Academy and Centre of Excellence).

Thus, there were now two parallel horse cultures in Ballyfermot:

• Individuals (predominantly youths) engaged in supervised equestrian training (and related social improvement schemes) in modern, subsidized equestrian facilities at Cherry Orchard, and

• A horse community whose young owners/keepers housed, grazed, manage, rode and drove horses in the urban spaces and endured periodic raids by contractors working under the direction of the Local Authority under the terms of the Control of Horses Act, 1996.

#### Fettercairn Youth Horse Project

This project runs in Tallaght, a built-up area of south County Dublin with generally similar demographics to Ballyfermot. The project was established in 1995 when funding was secured from Dublin South County Council and The Ireland Funds [9], and a facility developed which the local community felt they might use to house and keep their own horses in their own fashion. A block of 20 stables was commissioned on approximately 6 hectares of land. Over time it became apparent to project staff that the local horse culture remained largely unchanged - horses still roamed freely in the surrounding urban area - and the standards of horsemanship within the Fettercairn project did not approach equestrian norms.

Despite local resistance to change, at the time of writing some three quarters of the horses were now owned by the Fettercairn project rather than directly by the community. Consequently, the Centre's focus is now on changing the behaviour of those willing to engage with a structured programme, rather than accommodating those who wished simply to avail of a facility on their own terms. Riding lessons were provided at a subsidized rate; stable management and horse husbandry were taught and supervised; youths were accepted from such as the local drugs rehabilitation unit; and pupils have graduated to further training at RACE and the Irish Army Equitation School.

#### Meakestown Equestrian Facility

This project was developed as a green-field initiative in north-west Dublin during a time (the mid-2000s) when the nearby suburban areas of Finglas and Ballymun were the subject of major regeneration projects [10]. High-rise apartment blocks were being replaced by lower-density housing considered more in tune with the social needs of the community. Meakestown facility staff felt that that the equestrian project might represent a solution to two local horse 'problems':

• The area 'suffered' a high number of straying and unlicensed horses (in the sense of the Control of Horses Act, 1996), and

• It was felt that many of the horses presented at the monthly 'problematic' Smithfield market (see below) came from this horse population.

The Meakestown project was developed by Dublin City Council in conjunction with Ballymun Regeneration Ltd with a €3.5 million set up cost [11]. Architect-designed stables, meeting rooms, storage facilities, grazing and a horse exercise area were developed and a manager installed. However, members of the local community were permitted to move their own horses (and methods) onto the site, continuing to operate as before but in a subsidized facility. The Meakestown project seemed, at the time of visiting, to be experiencing some administrative difficulty.

#### Smithfield horse market

Smithfield market has a long-established association with horse ownership amongst the Traveller and inner city communities in Dublin, the communities which the social horse projects were largely set up to serve. The market is held in a built-up inner-city Dublin location on the first Sunday of every month. It is unregulated and horse numbers vary unpredictably from month to month (there are no pre-market entry requirements).

The market has been the subject of considerable discord between Dublin City Council and the local horse-owning community. A serious incident involving a run-away horse in 2002 led to Dublin City Council disassociating itself officially from the event (citing insurance difficulties). The market continued as before but with complaints increasing by such as the Dublin Society for the Prevention of Cruelty to Animals (DSPCA), members of the local business community, tourists and the general public. Attempts to close Smithfield market or move it to either Meakestown or Cherry Orchard were met by heavy resistance from regular attendees who have carried on, regardless of Dublin City Council and police stewarding, in the fair's traditional inner-city location and on its traditional calendar date. The fair has been the focus of ongoing negative media reporting of violent and unsocial behavior such that at the time of writing in 2011, further attempts are being made to close or relocate it.

#### Kilkenny Community Action Network (KCAN)

KCAN is a non-government organisation (NGO) funded by the Department of Community, Gaeltacht and Rural Affairs through the Local Development Social Inclusion Programme and managed by Pobal on behalf of government [12]. As one of its many initiatives aimed at addressing social exclusion of disadvantaged communities, it has sought to engage with adult male members of the Traveller community through the medium of horses. Grazing land was rented locally and a training programme instigated. At its peak, approximately twenty men (with forty horses) participated with a KCAN team comprising community workers, Local Authority Staff, an equestrian trainer and a veterinarian. KCAN project staff "recognized the effectiveness of using 'horse talk' as a forerunner to the introduction of other topics such as mental and physical health issues". Improvements in horse health and welfare were considered of secondary benefit. The next and seemingly natural step proposed for the project was the acquisition of a permanent home for the horse project. Pledges of substantial funding were secured to develop a permanent project with purpose-built facilities and grazing land permitting Traveller men to keep horses under supervision and engage in equestrian training. However, suitable land was not identified by the Kilkenny Local Authority at a critical stage in the project development, and the funding pledges were subsequently lost.

## Discussion

The Control of Horses Act, 1996 has proven to be a seminal piece of legislation regarding the keeping of horses. The Act was not devised to address equine welfare issues although there are limited circumstances in which authorised officers under the Act can directly insist that veterinary attention be sought and provided for equids. The Act appears as the dominant legislative instrument influencing how certain communities such as Travellers and inner city horse owners are expected to keep their horses. It has had a profound effect in areas and on populations where Local Authorities have chosen to implement it. This influence can be viewed in a most positive light in County Louth; however, the subcontracting model employed by most Local Authorities would appear to be a fire-fighting exercise at best. Additional concerns have arisen since the introduction on July 1^st ^2009 of EU Regulation 504/2008 (as implemented by SI 357 of 2011) regarding the identification of horses as microchip devices not linked with the issuing of horse passports were being inserted at horse pounds.

The routes of movement, sale and disposal of horses are not well documented or regulated in Ireland. The Tripartite Agreement permits free movement of horses between Ireland, the UK and France, without health certification, ostensibly only of non-slaughter, identified equidae accompanied by their passports. Horse-dealers take advantage of the lack of oversight and operate with impunity to free market principles exporting horses for slaughter but not openly declaring this intention. Proper oversight of horse movement would require extensive input at ferry ports and other border crossings with potentially major repercussions for the conduct of the normal business of trade in breeding and competition stock between the three countries concerned. Ferry ports officials currently do not examine horses or check that microchip numbers and horse markings corroborate with passport details; some do not record data for throughput. The gross numbers of horses moving out of Ireland (north and south) through those ports which recorded numbers can be seen to have remained relatively stable between 2006 and 2009 while the net export figure can be seen, from Table 2, to have almost doubled in the same period. The net trend is accounted for by a significant reduction in the movement of horses into Ireland. It is not possible to determine whether individual horses being imported are horses that have previously been exported, or to reliably quantify the total import/export numbers. The Irish Thoroughbred Breeders Society (ITBA), for example, claims that 6,222 horses were exported from Ireland in 2008 (4,171 to Great Britain) [13].

The knackery system was set up to manage the disposal of fallen farm stock with due regard to concerns for animal health and welfare and for the environment. The service was subsidised by the Fallen Animal Scheme until 2009 when this support was discontinued. Though not originally intended as a service to the equine industries, the Fallen Animal Scheme covered the cost of rendering and disposal (though not collection) of horse carcases and its withdrawal can only have had a negative effect on the numbers of horse carcases processed by this route. Knackeries can be seen from the enquiries conducted in 2007 not to deal with significant numbers of horse carcases (in comparison to production numbers [1]) although as there has been no requirement to record actual throughput, it must be acknowledged that the figures presented are an estimate only.

Statutory Instrument 612 of 2006 sets out the legislative position (as per EC Regulation 1774 of 2002) in the Republic of Ireland regarding the burial of carcases. A derogation exists permitted the disposal of pet animals, defined as 'any animal belonging to species normally nourished and kept, but not consumed, by humans for purposes other than farming', under license. This derogation is not normally felt to apply to horses but there must be concern that the numbers of horses buried in remote locations will increase as the cost of legitimate routes of disposal for horses excluded from the human food chain also increases. In Northern Ireland no co-ordinated system of knackeries for the disposal of horses exists; horses are often held to come within the definition of 'pet animal' as defined by the relevant legislation and thus on-farm burial is considered to occur with greater frequency than in the Republic of Ireland.

Disposal of horses through abattoirs for human food trade is a comparatively more lucrative method of disposal of horses for owners. Italy is Europe's largest market for horse meat and one where much of the lower quality product is further processed. There is major competition, however, in the marketplace from suppliers of horse carcasses in North and South America and Eastern Europe, and from the live horse trade. Live transportation for slaughter is driven by a cultural desire for horse meat from horses slaughtered locally and thus perceived to be local even if actually from horses imported live immediately prior to slaughter. France and Belgium represent added-value markets - there is a desire for higher quality unprocessed product. The major problems for Irish suppliers into the Continental market are that many Irish horses are of perceived non-meat breeds such as the Thoroughbred, Irish business operates in a high cost environment, there is a significant added cost associated with transport to the market place, and it may be difficult to secure payment for product.

In Ireland, horses are not generally bred for the meat trade and the horse slaughter business has largely been conducted in an unobtrusive fashion due to concerns that it is not a trade that the general public is likely to view in a favourable light. A growing issue is that many will have received medications that preclude them from entering the human food chain. Public health considerations drive a policy of strict oversight by DAFF and Local Authorities in Ireland. Strict control over the selection of appropriate horses with "clean" passports, which are not recorded as having received prohibited medications, means that his route is not open to many horse owners in the ROI.

From a welfare perspective, the humane destruction of unwanted horses at home (and subsequent disposal via knackeries) and at supervised abattoirs ought to be facilitated in preference to their movement over indeterminate time and distances via fairs, markets and dealers, which latter trade is likely to increase stress and therefore compromise horse welfare. This is, however, a complex argument and one easily misrepresented in the media. For example, the humane slaughter of horses at an approved abattoir and subsequent supply of skin-covered carcases (improving carnivore welfare) to Dublin zoo was described in one national newspaper as: *"Slow racehorses fed to the lions in Dublin zoo" *[14].

Social horse projects are a commendable attempt to engage locally with urban communities who wish to keep horses, serving the twin aims of engaging with authority-shy groups predominantly young males, and improving the local horse culture to the benefit of all. However, those whom the project aims to assist may themselves resist engagement as they perceive a different need to the project's stated aims. And these projects often suffer from the perception of low public good and therefore from resistance by such as local politicians who can exert downward funding pressure.

FAWAC is a non-statutory government advisory committee, established in 2002, which comprises representatives from stakeholder bodies such as farming and veterinary organisations, educational and scientific institutions, animal welfare charities and government departments. It issues guidance documents [8] and advisory position statements to the Minister for Agriculture on concerns relating to the welfare of farmed animals, which category has increasingly been considered to include horses [6]. In 2007, a sub-committee (the Equine Welfare Liaison Working Group) was established in response to the perception of a growing need to address the plight of unwanted horses. Members of FAWAC expressed concern for a perceived worsening of welfare conditions for horses on farms and at fairs and the need to improve existing routes for the humane disposal of unwanted horses. Members proposed that the correct identification of equidae receive appropriate legislative attention as being fundamental to achieving improvements in equine health and welfare. Advisory documents were issued to government, which, however, it is not statutorily obligated to accept and most likely views in the much wider context of animal health, agri-economics and the political reality.

Establishing a coordinated system for the registration of horses, transfer of ownership and monitoring of movement in and out of Ireland is essential, in the opinion of the authors of this paper, to safeguarding equine biosecurity and welfare in Ireland. Failure in this regard means that responsibility cannot be defined and traceability of horse movement in the face of contagious disease is extremely difficult. As per the European Communities (Equine) Regulations of 2011 (SI 357 enacted in July 2011), horse identification details will not, in the foreseeable future be centrally recorded in such as fashion that each animal can be traced from birth, from one owner/keeper to the next (as persons responsible for the animals' welfare) and to a humane endpoint.

## Conclusions

There is a huge variance in how the Control of Horses legislation has been employed across Local Authority areas in Ireland and there is thus a very real concern that pressure applied in one area simply leads to a movement of the problem elsewhere. Fundamental to an understanding of why there is insufficient co-ordination of routes for the proper, timely and humane disposal of horses is the lack of a comprehensive, integrated system for the registration, identification and tracing of equidae. And social horse projects, though laudable, suffer (as a means of improving horse welfare standards) from the difficulty that results (in terms of both human and horse welfare) are often intangible and long-term in nature. All of the above point to the need for a more centrally mandated and uniformly applied system of governance to promote the production, keeping and disposal of horses to a higher welfare standard [15].

## Competing interests

The authors declare that they have no competing interests.

## Authors' contributions

All authors contributed to the design of the study. JC gathered primary data under the supervision of VD and drafted the manuscript under the direction of SM. PW advised on research methods and AH on the final form of the manuscript. All authors read and approved the final manuscript.
